# Pharmacological intestinal obstruction because of calcium polystyrene sulfonate administration

**DOI:** 10.1093/jscr/rjae201

**Published:** 2024-04-04

**Authors:** Benjamin Thorpe, Salustiano Gonzalez Vinagre, Daniel Santos, Javier Caneiro Gomez, Manuel Bustamante Montalvo

**Affiliations:** General Surgery Department, Hospital Clinico Universitario de Santiago de Compostela, Santiago de Compostela, Spain; General Surgery Department, Hospital Clinico Universitario de Santiago de Compostela, Santiago de Compostela, Spain; Anatomical Pathology Department, Hospital Clinico Universitario de Santiago de Compostela, Santiago de Compostela, Spain; Anatomical Pathology Department, Hospital Clinico Universitario de Santiago de Compostela, Santiago de Compostela, Spain; General Surgery Department, Hospital Clinico Universitario de Santiago de Compostela, Santiago de Compostela, Spain

**Keywords:** cation exchange resins, hyperkalemia, intestinal obstruction, polystyrene sulfonic acid, renal insufficiency, chronic

## Abstract

Cation exchange resins are commonly used as treatment for hyperkalaemia in patients with chronic renal disease. There is a relation between cation exchange resins and the development of gastrointestinal adverse effects. A case of an intestinal obstruction at the terminal ileum is presented that underwent an ileocolic resection because of a critical stenosis of the intestine. The pathologist revealed abundant inflammatory cells together with deposits of calcium polystyrene crystals responsible of the intestinal obstruction. A rare cause of intestinal obstruction to bear in mind in chronically medicated patients with cation exchange resins.

## Introduction

Hyperkalaemia (HK) is one of the most common electrolyte disturbances in clinical practice [1]. Potassium-binding resins are widely used for the treatment of HK even though they have proven limited efficacy and bad tolerance [1, 2].

The use of cation exchange resins (CER), with or without sorbitol, is associated with gastrointestinal disturbances [3] in some cases as severe as colon necrosis or intestinal perforations. The most frequent site of damage is the colon but cases affecting the upper gastrointestinal tract have been described [4]. The adverse effects may not be common; nevertheless, the widespread use of CER may be exposing a large population to an unnecessary potential risk [3].

## Presentation of case

A 70-year-old woman was admitted to the emergency department of our hospital because of persistent abdominal pain, absence of bowl movement, together with vomiting of 24 h. She had previous diagnosis of chronic renal disease (CKD), ischemic heart disease, chronic gastritis, gallstones, chronic anemia, and myelofibrosis post-essential thrombocytosis JAK2V617F, currently in treatment with Busulfan and Hydrea. She was chronically medicated with allopurinol, omeprazole, acetylsalicylic acid, atorvastatin, hydroxychloroquine, dapagliflozin, alprazolam, duloxetine, bisoprolol, furosemide, acenocoumarol, and for over 2 months, she had been on treatment with oral calcium polystyrene sulfonate.

The patient arrived apiretic, with normal tensional values and tachycardia (112 bpm). During the physical examination, we found a distended, tympanic but soft abdomen with localized pain and abdominal guard after compression on the right lower quadrant of the abdomen.

**Figure 1:**
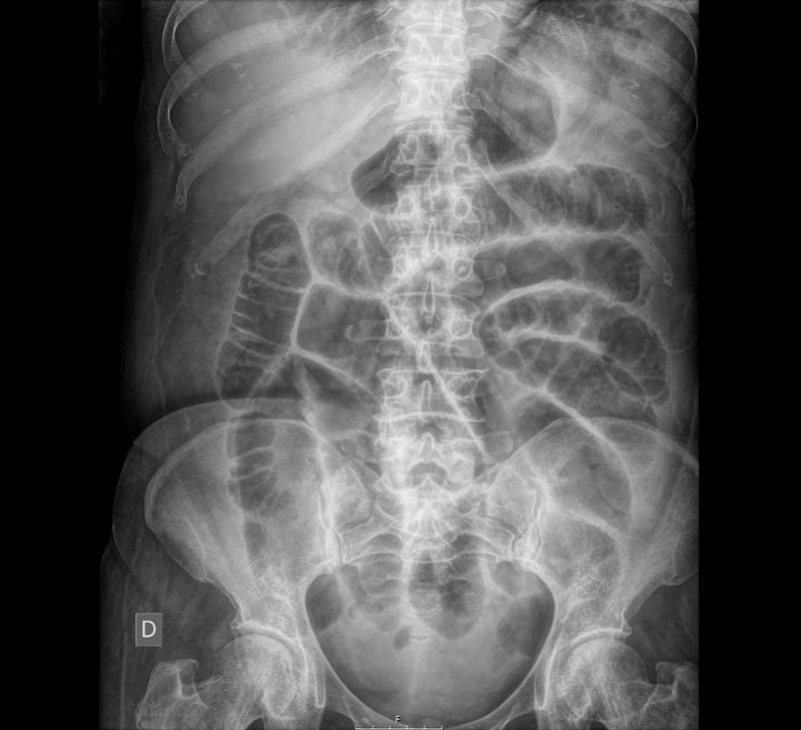
Abdominal X-ray: generalized dilatation of the small intestine without distal air in the colon or the rectum.

**Figure 2:**
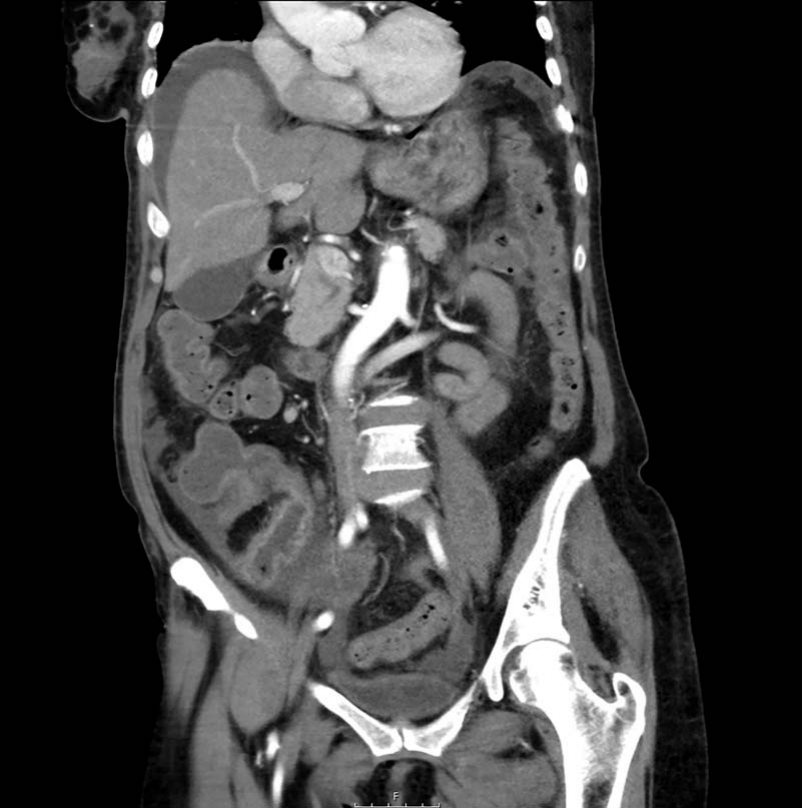
CT scan: sudden change of caliber at the terminal ileum together with signs of intestinal suffering.

The laboratory results rebelled leukocytosis (15.23x10e3), anemia (9.6 g/dl), neutrophilia (80.4%), band neutrophils in peripheral blood (15%), an elevated C reactive protein (20.62 mg/dl), and procalcitonin (3.92 ng/dl) values (Figs 1 and 2).

**Figure 3:**
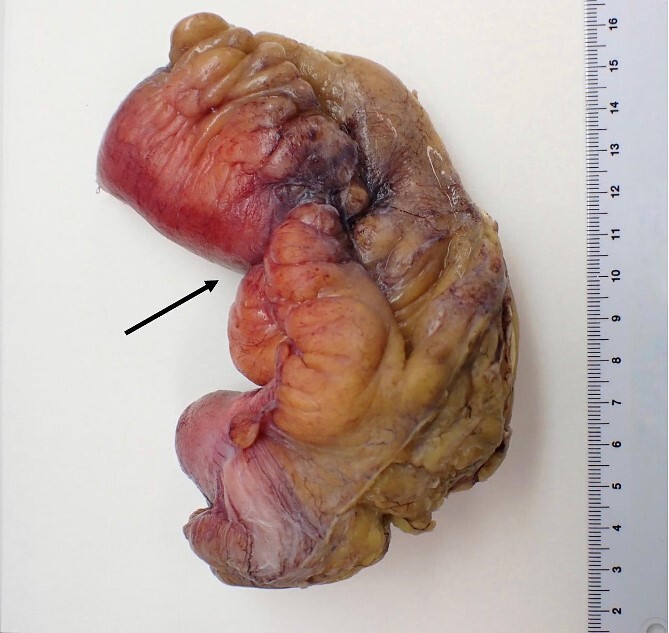
Macroscopic pathological examination: mesenteric hypertrophy surrounding the mesoileum causing stricture of the intestinal lumen.

**Figure 4:**
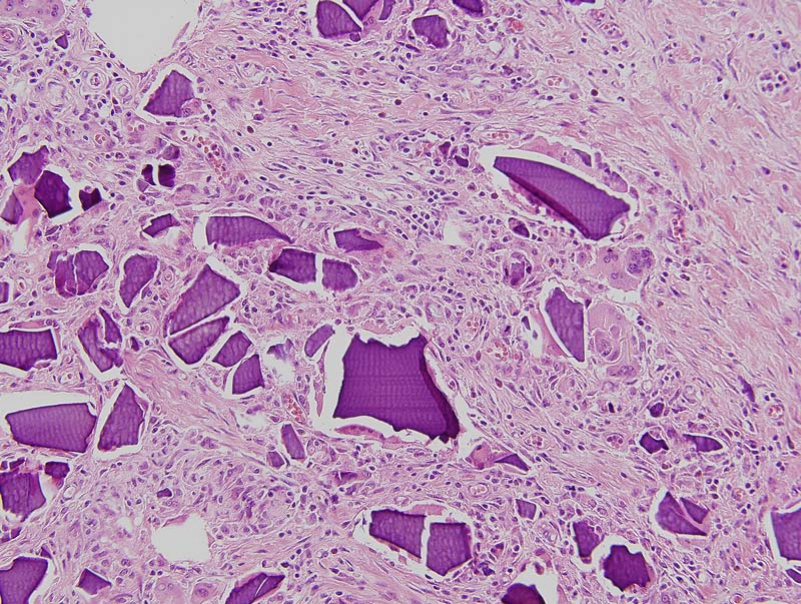
Microscopic pathological examination: abundant calcium polystyrene sulfonate crystals along the intestinal wall surrounded by intense inflammatory infiltrate.

**Figure 5:**
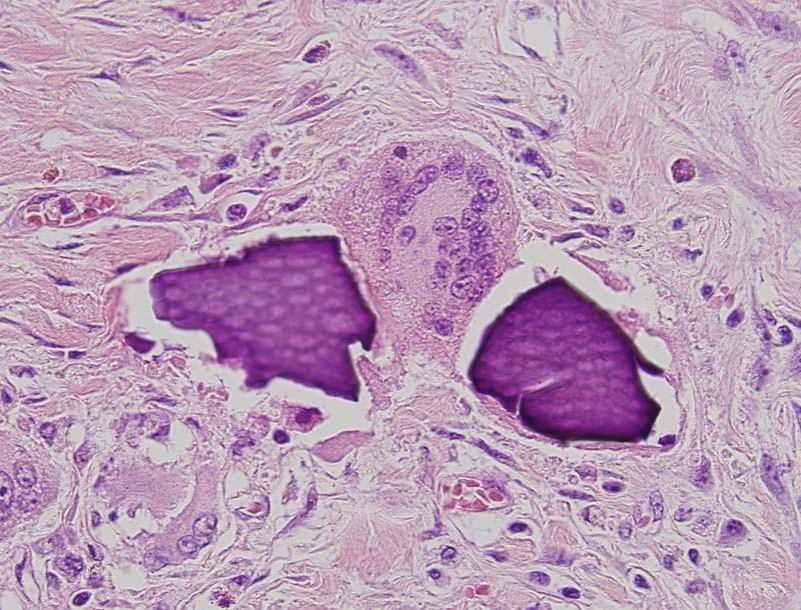
Multinucleate giant cell trying to phagocytize calcium polystyrene crystals.

An urgent laparotomy exposing the terminal ileum was performed, signs of intestinal suffering were evident in relation with a thickening and a retraction of the mesoileum at the terminal ileum responsible for the small bowl obstruction. An ileocecal resection with immediate side-to-side ileocolic anastomosis was performed (Fig. 3). Microscopic pathological examination: abundant calcium polystyrene sulfonate crystals along the intestinal wall surrounded by intense inflammatory infiltrate (Figs 4 and 5).

The patient was discharged on the 17th day of hospitalization with restoration of the intestinal transit. One month after the patient suffered an ischemic stroke on the right anterior cerebral artery with good evolution but diminishing her previous quality of life.

## Discussion

HK is the most frequent electrolyte disturbance present in the daily medical practice. It increases the chances of sudden death, as a consequence of the appearance of fatal cardiac arrhythmias. There are many risk factors for HK including cardiovascular diseases, diabetes mellitus, acute or chronic kidney disease (CKD), and the administration of drugs that inhibit the renin–angiotensin–aldosterone system (RAAS) [1, 2].

Regarding the treatment of HK, we encounter dietary potassium restrictions, administration of diuretics, withdrawal or dose reduction of RAAS inhibitors, and the usage of CER. Older CER, calcium polystyrene sulfonate, and sodium polystyrene sulfonate have proven limited efficacy and multiple side effects, being the digestive system the most common target [2]. Recent CER, patiromer and sodium zirconium cyclosilicate, seem to show better long-term potassium blood level control, they allow the maintenance of concomitant prescription of RAASIs and present limited adverse effects [2, 5]. The aim of these new CER is to delay the need of renal replacement therapy [2].

The administration of older CER has been associated with several gastrointestinal side effects that vary from nausea, vomiting, and diarrhea to less common but more damaging side effects such as severe intestinal ulcers, bowel obstruction, intestinal necrosis, or colonic perforations [2, 6]. Certain risk factors seem to enhance the damage of CER over the digestive system, some of which are CKD, solid organ transplantation, advanced age, intestinal fragility, or constipation [6, 7]. The colon remains the most frequent site of injury [4, 7]. Harel *et al*. describe that only 30% of the gastrointestinal lesions appear on proximal locations of the gastrointestinal tract including the esophagus, the stomach, the duodenum, and the small bowel. Nonetheless the majority of these proximal lesions revealed concomitant colonic injuries [3, 8].

Murakami *et al*. performed a retrospective study comparing 61 end-stage renal disease patients developing acute peritonitis and reviewing retrospectively if they had been treated with CER. The study describes 35 patients receiving oral CER preoperatively and 26 patients that did not receive CER at all. None of these patients received concomitant treatment of CER with sorbitol. The incidence of CER-related intestinal necrosis was ~0.57% when administered alone and 1.8% when administered together with sorbitol [7].

Because of the frequency of adverse events related to the usage of CER with 70% sorbitol, the United States Food and Drug Administration removed the recommendation of the conjoint administration of sorbitol with CER in 2009 [4]. Thenceforth more cases of severe gastrointestinal damage have been reported in patients receiving CER in monotherapy [3, 7, 8].

Dosage of CER responsible for gastrointestinal disturbances remains undefined, varying from patient to patient. It has been suggested that intestinal complications are less likely to appear in patients receiving a lower dose of CER. Studies struggle to collect data regarding the dose and the route of administration of CER in each patient, hindering the dose-response association [8, 9].

Different hypotheses suggest the mechanism by which CER contribute to gastrointestinal injuries. In vitro studies propose that CER crystals when in contact with intestinal epithelial cells may trigger inflammation processes that result in necrosis of the gastrointestinal mucosa, more so, in patients with previous preexisting chronic comorbidities that can alter the intestinal epithelial cell barrier. Understanding that the translocation of intestinal microbiota because of cell barrier dysfunction will favor a inflammatory response causing necrosis, ulceration, or even perforation of the gastrointestinal tract [4, 10]. This mechanism is also seen in patients treated with sevelamer, an anion exchange resin used for the treatment of hyperphosphatemia with reported cases of sigmoid colon perforation showing sevelamer crystals at the perforation site [10, 11].

Physicians prescribing CER have to bear in mind the possibility of adverse gastrointestinal effects, especially in critically ill patients with important risk factors. Once the diagnosis of CER-related gastrointestinal injuries has been established, CER should be removed to prevent the patient from future episodes.

## Conflict of interest statement

None declared.

## Funding

None declared.
